# Individual Diameter Growth Modeling of *Terminalia alata* (B. Heyne. ex Roth) in Terai Arc Landscape of Nepal

**DOI:** 10.1155/sci5/5518089

**Published:** 2024-12-19

**Authors:** Pratima Gautam, Rajeev Joshi, Santosh Ayer, Jeetendra Gautam, Kishor Prasad Bhatta, Prakash Lamichhane

**Affiliations:** ^1^Department of General Forestry, Agriculture and Forestry University, Hetauda, Makwanpur 44107, Nepal; ^2^Department of Silviculture and Forest Biology, College of Natural Resource Management, Faculty of Forestry, Agriculture and Forestry University, Katari, Udayapur 56310, Nepal; ^3^Department of Forest Biometrics, College of Natural Resource Management, Faculty of Forestry, Agriculture and Forestry University, Katari, Udayapur 56310, Nepal; ^4^Department of Forest Survey and Engineering, Agriculture and Forestry University, Hetauda, Makwanpur 44107, Nepal; ^5^Department of Forest Research, Research and Development Centre (RDC), Kathmandu 44600, Nepal; ^6^Department of Forest Biometrics, Forest Research and Training Centre, Babarmahal, Kathmandu 44600, Nepal

**Keywords:** diameter measurement, forest inventory, individual growth modeling, regression models, Terai Arc Landscape

## Abstract

The development of a model is highly crucial in cases where there are intricate geographical features, and conducting a forest inventory is both time-consuming and expensive, requiring significant manual effort for measurement. Acquiring reliable data regarding the forest's condition and future progression is essential for making informed decisions about its management. Therefore, this research aimed to create an individual tree diameter growth model specifically for *Terminalia alata* (B. Heyne. ex Roth). This study was conducted in Terai Arc Landscape of Nepal, encompassing 14 districts in the Terai and Chure regions of Nepal. Individual tree data (diameter at breast height, tree height, crown height, crown cover, longitude, and latitude) from three different time periods (2011, 2017, and 2022) were obtained with 673 sample plots maintained for forest research assessment by Government of Nepal, and annual diameter growth was estimated. Multiple linear, linear mixed, and generalized additive models were employed to fit the growth modeling for individual tree diameter growth of *T. alata*. We observed higher mean diameter growth rates in 0–25 cm and 101–125 cm tree diameter classes (0.318 cm·yr^−1^). There were significant differences in diameter growth across tree quality classes, but no significant differences due to crown classes were observed. Although the generalized additive model (Adj. *R*^2^ = 0.32) performed better than the linear mixed model (adj. *R*^2^ = 0.23) and the multiple linear model (adj. *R*^2^ = 0.03), it still explained only a small proportion of the variance in diameter growth. This suggests that other factors, such as unmeasured environmental variables, biotic interactions, or complex nonlinear relationships, may play a significant role in explaining the variation. In addition, the low *R*^2^ values indicate that the models may need further refinement, possibly by incorporating interaction terms, random effects, or other possible nonlinear approaches. Future research should also consider the potential influence of spatial or temporal heterogeneity on the growth dynamics.

## 1. Introduction

Growth refers to the increase in size of one or more individuals within a forest stand over time [1], while a model is a simplified representation of a certain aspect of reality [2]. Growth models are mathematical representations used to simulate the growth processes of trees and forests [3]. These models typically involve a combination of mathematical equations, numerical values, and logical connections that describe how trees grow in response to various environmental factors [4]. Forest growth models describe how the size of a tree, another tree attribute, or a stand-level variable changes with age [5]. These models are used to evaluate and estimate various dynamics in forest stand development, including species composition, site characteristics, tree competition status, and silvicultural management [6–8].

While many tree-based models employ similar general approaches, the specific functional forms may differ slightly. Most models use tree diameter as the primary unit of growth measurement, although some incorporate tree height, bole diameter at different heights, and crown size. Foresters are typically interested in assessing merchantable timber production at specific sites under defined environmental and silvicultural conditions. Forest growth equations are particularly focused on forest production within prescribed site and management conditions rather than community dynamics. They are highly valuable in determining optimal rotation and thinning schedules, as well as plant spacing designs to maximize wood production under specific silvicultural treatments [9].

Estimating tree volume is crucial in forest management for assessing current and future forest stocks [10]. However, directly measuring volume in the field is time-consuming and impractical. Models or mathematical functions are necessary to estimate volume using measurable characteristics such as height, diameter, and tree shape [11]. Individual tree diameter and height growth models are integral components of forest growth and yield frameworks, serving as essential tools for sustainable forest management planning [12]. While both height and diameter are commonly measured in routine forest assessments, measuring tree height can be impractical due to its time-consuming nature, cost, and potential for decreased accuracy [13–15]. Measuring diameter at breast height (DBH), on the other hand, is often quicker, more accurate, and more affordable. A volume table generated using a model with DBH as the sole predictor variable is especially useful for rapid timber inventories, requiring only species identification and DBH measurement [15–17]. Therefore, DBH-based growth models serve as practical and efficient tools for estimating tree volume and predicting tree height in forest management.

To project the future productivity of plantation forests, forest managers rely on a variety of growth models [18]. While stand-level growth models are suitable for predicting the development of forests with uniform age structures, the dynamics of mixed or uneven-aged forests require a different approach—individual tree growth models [3]. Unlike stand-level models, which provide an overview of the forest as a whole, individual tree growth models take a bottom-up approach. They start by examining the growth of each individual tree and then integrate these insights to understand how the entire stand evolves over time [19]. When comparing growth simulations between pure even-aged forests and mixed uneven-aged forests, the complexity and scope of the models increase significantly [20]. In mixed forests, where tree ages and species composition vary, individual tree growth models offer a more comprehensive understanding of growth patterns. This shift toward individual tree models reflects a deeper understanding of forest ecosystems and allows for more accurate predictions of future forest dynamics [21]. Consequently, individual tree growth models are becoming increasingly popular in forest management practices, offering valuable insights into the management of diverse forest landscapes [22].

*Terminalia alata* B. Heyne. ex Roth, a member of the Combretaceae family, is a tree species commonly found in tropical and subtropical forests. It thrives at elevations up to 1400 m and is native to several countries including India, Myanmar, Nepal, and Thailand [23]. Of the total species measured in the forests of Nepal, 14 species make up more than 1% of the total trees, and *T. alata* is one of them [24]. We can say it is one of the major tree species of Nepalese forest with ecological, economic, and cultural benefits. Ecologically, it aids in soil conservation, supports biodiversity, and helps mitigate climate change through carbon sequestration. Economically, it provides durable timber, used in construction, furniture, tool handles, and underwater purposes [25]. The wood, with a density of about 1040 kg/m^3^ at 12% moisture content, is also valued for attractive veneer [23]. In addition, its leaves are used as fodder in Nepal, and the bark and fruit yield pyrogallol and catechol for dyeing and tanning leather. Medicinally, bark is used to treat diarrhea [23] and can be a source of oxalic acid [26]. Given its importance, studying *T*. *alata* and developing individual growth models is essential. Developing individual growth models for *T. alata* would provide valuable insights into its growth patterns, environmental interactions, and potential responses to changing conditions. Previous research in Nepal has primarily focused on individual tree growth modeling for species such as *Shorea robusta* [27], *Pinus roxburghii* [28, 29], *Pinus wallichiana* [30], *Alnus nepalensis* [31], *Castanopsis indica* [32], and *Cedrus deodara* [29]. However, there remains a notable gap in research concerning *T. alata* which our study aimed to fill it. Therefore, this study aimed to model the individual tree growth of *T. alata* using diameter measurement at different time interval in Terai Arc Landscape (TAL) of Nepal. The specific objectives of this study were (i) to analyze the diameter distribution of selected trees, (ii) to evaluate the relationship between crown class and quality class with diameter growth, and (iii) to determine the best-fitting diameter growth model for *T. alata* by comparing the multiple linear model, linear mixed model, and generalized additive model.

## 2. Materials and Methods

### 2.1. Study Area

**Figure 1:**
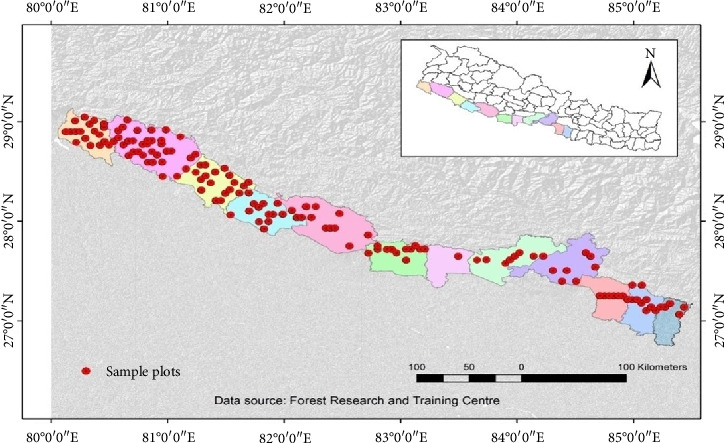
Study area map of TAL Nepal showing established 673 sample plots.

This study was conducted in the TAL region of Nepal, which spans from the Bagmati River in the east to the Mahakali River in the west, and from the Indian border in the south to include the central and western parts of the Chure range of Nepal (Figure 1). TAL covers an area of 23,199 sq·km. which encompasses 14 districts of the Terai region of Nepal (Rautahat, Bara, Parsa, Makwanpur, Chitwan, Nawalparasi, Rupendehi, Palpa, Kapilbastu, Dang, Banke, Bardiya, Kailali, and Kanchanpur) [33]. TAL represents around 15% of Nepal's total land area and 20% of its national forest area. About 54% of the total area of TAL is covered by forest which mainly constitutes of broadleaved mixed forest, *Shorea robusta* forests, and grasslands [34]. The TAL region includes five protected areas and their respective buffer zones, namely, the Parsa National Park, Chitwan National Park, Bardia National Park, Banke National Park, and Shuklaphanta National Park [35]. The dominant tree species found in this area are *Shorea robusta*, *Terminalia alata*, and *Lagerstroemia parviflora* [36].

### 2.2. Sampling Design and Data Collection

**Figure 2:**
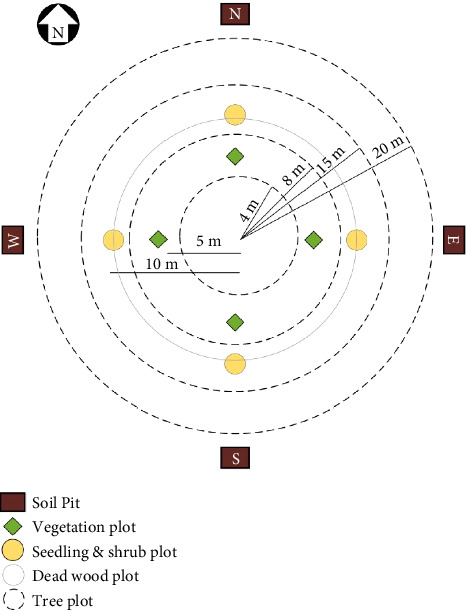
Layout of concentric sample plots for trees and subplots for soil and smaller vegetation (source: [24]).

The Department of Forest Research and Survey (DFRS) (now Forest Research and Training Center) conducted Forest Resource Assessment (FRA) between 2010 and 2014 utilizing a stratified systematic cluster sampling design nationwide [24]. Stratification involves grouping the population into homogeneous subgroups before sampling, while systematic cluster sampling is employed within each stratum to enhance the representativeness of the sample and reduce sampling error. Clusters, which are groups of sample plots, are used when the population can be divided into separate groups. The FRA implemented a two-phase cluster sampling method: in the first phase, a 4 × 4 km grid was established, and clusters of plots were established at each grid point. In the second phase, a subsample of clusters was selected for field measurements. In the Terai physiogeographic zone, including the Inner Terai, there were only four sample plots per cluster. For inventory purposes in natural forests, concentric circular sample plots (CCSPs) were recommended for tallying trees due to their reduced edge effects compared to square or rectangular plots and the ease of identifying border line trees. Each plot was considered a permanent plot. The CCSP utilized in the FRA consisted of four circular plots (Figure 2). The data for this study consisted of information from 673 sample plots (Figure 2) gathered at three different time periods (2011, 2017, and 2022). Sample plot coordinates were collected using a handheld GPS, while precise locations were determined with a differential GPS (DGPS). Tree height measurements were obtained using a Vertex IV and Transponder T3 whereas *D*-tape was used to measure DBH. Similarly, crown cover was estimated with a spherical densiometer.

### 2.3. Models

Linear mixed models are a type of regression analysis that combines fixed effects (predictors) with random effects (grouping variables) to model the relationship between variables [37]. Linear mixed models could be applied to analyze hierarchical data structures commonly found in forestry, such as longitudinal data or data collected from different spatial scales within a forest ecosystem [38]. For example, linear mixed models could be used to model the growth trajectories of individual trees over time, while accounting for factors such as site conditions, management practices, and spatial autocorrelation [39]. This approach allows for the incorporation of both fixed effects (e.g., management interventions) and random effects (e.g., site-specific variability) in the analysis, providing insights into how different factors influence forest growth and development [38]. Multiple linear models are another form of regression analysis that models the relationship between a single dependent variable and two or more independent variables [40]. Multiple linear models could be employed to investigate the relationships between multiple predictor variables (e.g., soil properties, climate variables, and stand characteristics) and a single outcome variable of interest (e.g., tree volume, biomass, and species diversity) [41]. While linear models are valuable for understanding relationships that are expected to be linear, generalized additive models offer greater flexibility by allowing the modeling of nonlinear relationships between predictors and the response variable. Therefore, we also adopted the generalized additive model along with the multiple linear model and linear mixed model. The multiple linear model (equation (1)) assumes a linear relationship between the dependent variable and several independent variables, but it does not account for random effects. This model assumes that all observations are independent and that the relationship between predictors and the outcome is the same across all levels of random factors (e.g., tree quality class, plot). The linear mixed model (equation (2)) extends the multiple linear model by incorporating random effects to account for group-level variations, such as tree quality class or plot, which introduces nonindependence among observations within the same group. In the generalized additive model, the relationship between the response and predictors is not assumed to be linear. Instead, each predictor is modeled using a smooth function (often cubic splines) (equation (3)). In addition to smooth terms, the generalized additive model can include parametric terms (e.g., linear terms or factors). These are included to model the effects of categorical variables (such as a factor like “year” or “site”) or other variables that may have a fixed, linear effect on the response variable.

(1) $$\begin{array}{c} Y=\beta_{0\text{⁣}}+\beta_{1\text{⁣}}X_{1}+\beta_{2}X_{2}\ldots \ldots \ldots +\beta_{p}X_{p}+\in , \end{array}$$

where *Y* is the dependent variable (e.g., diameter growth), *β*_0⁣_ is the intercept, *β*_1_,  *β*_2_,…, *β*_*p*_ are the coefficients for the independent variables *X*_1_, *X*_2_,…,  *Xp*, and ∈ is the error term.

(2) $$\begin{array}{c} Y_{ij}=\beta_{0\text{⁣}}+\beta_{1\text{⁣}}X_{1ij}+\beta_{2}X_{2ij}\ldots \ldots \ldots +\beta_{p}X_{pij}+\mu_{j}+\in_{ij}, \end{array}$$

where *Y*_*ij*_ is the dependent variable for observation *i* in group *j*, *β*_0_, *β*_1_,…, *β*_*p*_ are the fixed effect coefficients (similar to the multiple linear model), *μ*_*j*_ is the random effect for group *j* (e.g., tree quality class, plot) which allows the intercept to vary across groups, and ∈_*ij*_ is the error term for observation *i* in group *j*.

(3) $$\begin{array}{c} Y_{ij}=\beta_{0\text{⁣}}+f_{1}\left(X_{1ij}\right)+f_{2}\left(X_{2ij}\right)\ldots \ldots \ldots .+f_{p}\left(X_{pij}\right)+\mu_{j}+\in_{ij}. \end{array}$$

*Y*_*ij*_ is the response variable for the *i*th observation in the *j*th group or cluster. *β*_0_ is the intercept term (fixed effect), *f*_1_(*X*_1*ij*_), *f*_2_(*X*_2*ij*_),…, *f*_*p*_(*X*_*pij*_) are the smooth functions applied to the predictor variables *X*_1*ij*_, *X*_2*ij*_,…, *X*_*pij*_. These functions model nonlinear relationships between the predictors and the response variable. The smooth functions *f*_1_, *f*_2_,…*f*_*p*_ are typically modeled using splines or other smoothers in a GAM framework; μ_j_ is the random effect for the *j*th group or cluster, which models group-level variability, and ∈_*ij*_ is the residual error term for the *i*th observation within the *j*th group.

### 2.4. Data Analysis

The dataset comprises potential predictors, encompassing individual tree attributes, plot-level attributes, and site attributes. The two of the predictors initially existed as nonordinal categorical variables, while the remaining predictors were numeric. The first step involved consolidating the data from repeated measurements into a unified growth data frame, which calculated the mean annual growth of all trees that were alive across all measurements. The estimation of annual diameter growth was carried out as follows:

(4) $$\begin{array}{c} 1\text{st annual diameter growth }\left(1\text{st period}\right)=\left(\frac{\text{DBH }2017-\text{DBH }2011}{\text{no}.\text{ of years}}\right), \end{array}$$

(5) $$\begin{array}{c} 2\text{nd annual diameter growth }\left(2\text{nd period}\right)=\left(\frac{\text{DBH }2022-\text{DBH }2017}{\text{no}.\text{ of years}}\right), \end{array}$$

where DBH 2011 = DBH recorded in the year 2011, DBH 2017 = DBH recorded in the year 2017, and DBH 2022 = DBH recorded in the year 2022.

The effect of crown class and quality class on diameter growth was examined using a nonparametric test, that is, the Kruskal–Wallis rank sum test [42]. Pairwise comparisons between the classes were conducted using the Dunn test [43]. The aggregated data were processed using *R*-script in the *R*-program, utilizing various packages including *tidyverse*, *patchwork*, *lme4*, *ggplot2*, and *dplyr*. The two models, namely, the multiple linear model and linear mixed model, were employed to fit the growth modeling for individual tree diameter of *T. alata*. The performance of the DBH growth equations was assessed through numerical and graphical analysis of residuals. The coefficient of determination (*R*^2^) was calculated to assess the goodness of fit of a both models. The formula for this statistic is as follows:

(6) Adj.R2=1−∑i=1nyi−yi^2∑i=1nyi−yi¯2.

## 3. Results

### 3.1. Descriptive Statistics of Tree-Level and Plot-Level Attributes

**Figure 3:**
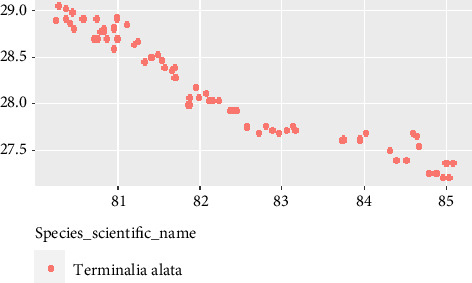
The location of the trees (*X*-axis and *Y*-axis represent longitude and latitude, respectively).

The descriptive statistics for the individual tree-level and plot-level attributes utilized in this study are presented in Table 1. The sample trees had a mean DBH of 38.8 cm, with a minimum and maximum DBH of 5.1 and 119 cm, respectively. The standard deviation and variance for DBH were calculated as 23.9 and 571.1, respectively. The height of the trees exhibited a standard deviation of 9.1, a variance of 82.6, and mean height of 19.3 m. Furthermore, the mean elevation of the plots, represented by DEM, was 477.4 m. However, the elevation data exhibited considerable variability, as evidenced by a standard deviation of 301.5 and variance of 90.69. The mean, standard deviation, and variance of longitude for the plots were 81.9°, 1.4, and 1.9, respectively, while for latitude, the corresponding values were 28.3°, 0.5, and 0.3, respectively. The latitude coverage of the plots extends from Eastern Rautahat to Western Kanchanpur, indicating a wider range. However, the longitude coverage is narrower as the plots are limited to the Terai and Chure regions of the country (Figure 3).

The assessment of crown class and quality class distribution showed that the majority of trees were in quality class 1, with 281 dominant and 55 codominant trees (Table 2). The quality class 2 had a notable number of dominant and codominant trees as well, while quality class 3 included a higher proportion of broken trees. In total, the dataset comprised 525 trees across all quality and crown classes (Table 2).

### 3.2. Annual Diameter Growth of Tree

The annual DBH growth (cm·yr^−1^) for the first and second periods showed some variation (Table 3). For the first period, the growth ranged from −0.03 to 1.86 cm·yr^−1^, with a median of 0.23 cm·yr^−1^ and a mean of 0.29 cm·yr^−1^. In the second period, growth ranged from 0.02 to 1.60 cm·yr^−1^, with a median of 0.22 cm per year and a mean of 0.29 cm per year. The mean DBH growth rates were similar across both periods, with the first period showing a slightly wider range of growth rates.

### 3.3. Diameter Growth by DBH Class

While analyzing the DBH growth of individual trees categorized into five different diameter classes: 0–25, 25–50, 50–75, 75–100, and 100–125, higher concentration of trees was observed in the 26–50 cm DBH class, followed by the 0–25 cm class (Table 4). In contrast, the 76–100 cm and 101–125 cm classes show relatively low tree counts. Among the different DBH classes, the 0–25 and 101–125 cm classes exhibit the highest mean diameter growth rates of 0.318 cm·yr^−1^. In contrast, the 26–50 and 76–100 cm classes show slightly lower growth rates, with 0.263 and 0.281 cm·yr^−1^, respectively. The 51–75 cm class falls in between these ranges with a mean growth rate of 0.311 cm·yr^−1^ (Table 4).

### 3.4. Diameter Growth by the Crown Class

**Figure 4:**
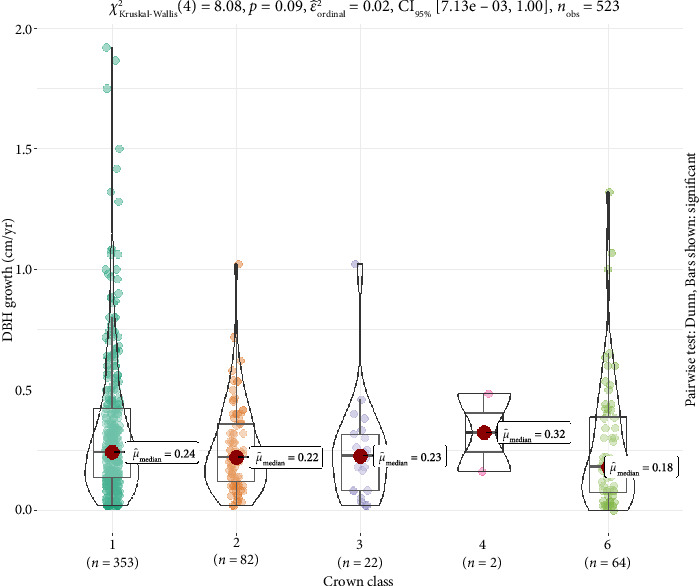
Annual DBH growth in each crown class. *X*-axis represents crown class and *Y*-axis represent DBH growth (cm·yr^−1^).

For DBH, crown class 1 exhibited the largest mean diameter (47.37 cm) with a wide range (5.3–114.8 cm), while crown class 3 had the smallest mean diameter (13.16 cm) (Table 5). The tree heights varied significantly, with crown class 1 averaging 23.25 m and crown class 6 the shortest at 8.79 m. Similarly, the mean growth rate was highest in crown class 1 (0.303 cm·yr^−1^) and lowest in crown class 3 (0.181 cm·yr^−1^) (Figure 4).

This study found no significant difference between the median of DBH growth across crown class (Figure 4). Although, the median value (μ^) was found to be higher in crown class 4, followed by crown class 1 and 3, in contrast, the 2^rd^ crown class (codominant) and 6^th^ crown class (broken) had relatively lower median values.

#### 3.4.1. Diameter Growth by the Quality Class

Quality class 1 trees exhibit the highest mean DBH at 46.32 cm, ranging from 5.2 to 118.6 cm, with a standard deviation of 23.08 cm (Table 6). In contrast, quality class 3 trees have the smallest mean DBH of 17.5 cm, ranging from 5.1 to 113.4 cm, with a standard deviation of 17.72 cm. Heights also vary significantly, with quality class 1 averaging 23.03 m and quality class 3 the shortest at 8.09 m. Growth rates are consistent across classes, with an overall mean of 0.29 cm/year (Figure 4).

**Figure 5:**
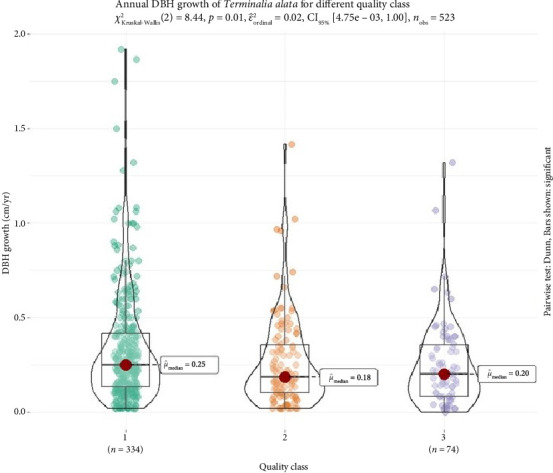
Annual DBH growth in each quality class. *X*-axis and *Y*-axis represent quality class and DBH growth (cm·yr^−1^). The “*n*” value represents the number of trees within each quality class.

We found significant differences between median of BH growth across quality class (Figure 5). The higher median value of DBH growth was observed in quality class 1 followed by quality classes 3 and 2.

### 3.5. Growth Model as Functions of Available Variables

#### 3.5.1. Multiple Linear Model

The multiple linear model demonstrated a statistically nonsignificant (*p* > 0.05), yet moderate, proportion of variance explained, with an R-squared value of 0.03, *F* statistics (7.372), and d*f* = 1.742 (Tables 7 and 8) The adjusted *R*-squared value was 0.01. In analyzing the impact of various factors on the growth rate, it was found that the DBH had a statistically significant and negative effect (*p* = 0.0254 < 0.05). On the other hand, the height, crown height, crown cover, DEM, latitude, and longitude showed a statistically nonsignificant effect on the growth rate.

#### 3.5.2. Linear Mixed Model

The linear mixed model demonstrated significant explanatory power, with a conditional *R*^2^ of 0.235, and the marginal *R*^2^ for fixed effects alone was 0.032. The effect of DBH was found to be statistically significant and negative (*p* < 0.05); however, tree height, crown height, crown cover, DEM, latitude, and longitude showed statistically nonsignificant effects on growth (*p* > 0.05) (Tables 9 and 10).

#### 3.5.3. Generalized Additive Model

In the generalized additive model for diameter growth, significant predictors include crown cover, height, DBH, and elevation, all showing substantial nonlinear effects on growth (Table 11). Crown cover, for example, has an effective degrees of freedom (edf) of 7.969 with a *p* value of 1.23e − 05, indicating a complex relationship with growth. Similarly, DBH is highly flexible (edf = 8.951, *p* < 2*e* − 16), which indicates growth rates vary considerably at different DBH levels. Dem (edf = 8.322, *p* = 0.0013) also significantly influences growth in a nonlinear manner. The random plot effect was not significant (*p* = 0.1872), implying plot-level variation has limited impact on growth in this model. Overall, the model explains 36.6% of the deviance in diameter growth with an adjusted R-squared of 0.326 (Table 12).

## 4. Discussion

### 4.1. Diameter Growth of *T*. *alata* Trees

The annual DBH growth rates observed in the first and second periods of the study reveal important insights into tree growth dynamics. In the first period, DBH growth ranged from −0.03 to 1.86 cm·yr^−1^ and 0.02 to 1.60 cm·yr^−1^ in second period (Table 3). These growth rates fall within the typical range observed by Lieberman et al. [44] and da Silva et al. [45] in tropical forest ecosystems. Despite the slight differences in the range of growth values between the two periods, the similarity in median and mean growth rates suggests that, on average, the trees were growing at comparable rates across both periods. However, the wider spread of growth values in the first period, with some trees showing negative growth, indicates that certain trees might have been in less favorable microenvironments, experiencing stress or limited resources. The highest mean DBH growth was found in both the smallest (0–25 cm) and largest (101–125 cm) DBH classes, with a growth rate of 0.318 cm per year (Table 4). This is somewhat surprising for the larger DBH class, as one might expect slower growth in mature trees. However, larger trees may still exhibit relatively high growth rates due to their established root systems, favorable growing conditions, or less competition for space and light in certain forest environments. Conversely, the 26–50 cm DBH class exhibited a lower mean growth rate of 0.263 cm·yr^−1^, which can be attributed to trees transitioning from juvenile to mature phases, a period when growth typically slows due to increased competition for resources. Similarly, the 76–100 cm class, with a growth rate of 0.281 cm·yr^−1^, shows a continued decline in growth as trees mature and begin to compete more for light, nutrients, and space. The concentration of trees in the 26–50 cm DBH class reflects the common structure of a forest stand, where younger and mid-aged trees dominate. The lower tree counts in the larger DBH classes indicate that larger trees are fewer in number due to factors such as natural mortality or thinning [46]. These results suggest that the forest is in a transitional phase, with younger trees growing rapidly while the older trees, although fewer, are still contributing to the overall growth. Our results align with the general finding that smaller trees have higher growth rates, which is consistent with findings from Nabeshima, Kubo, and Hiura [47], Rüger et al. [48], and Forrester [49] who observed faster growth in younger trees and slower growth in older, larger trees. The results also reflect typical forest dynamics, where thinning and natural mortality reduce the number of trees in the larger DBH classes over time [46].

The analysis of DBH growth by crown class revealed significant differences in growth rates across the various crown classes (Table 5, Figure 4). Trees in the dominant crown class, which have access to more sunlight and less competition, exhibited the highest mean diameter growth rate of 0.320 cm per year. This is expected as dominant trees generally have better access to resources such as light, water, and nutrients, leading to faster growth compared with trees in more shaded positions [50, 51]. On the other hand, codominant trees showed a lower mean growth rate of 0.274 cm per year, which is indicative of the competition they face for light and space (Table 5). Codominant trees are often partially shaded by neighboring dominant trees, leading to slower growth compared with their dominant counterparts [52, 53]. Intermediate and suppressed trees, which are generally more shaded and have limited access to resources, exhibited the lowest growth rates, with values of 0.242 cm per year and 0.215 cm per year, respectively (Table 5). These growth patterns reflect the fact that suppressed trees are often in more densely populated areas, where competition for light and space is intense, resulting in slower diameter growth [53]. These findings are consistent with studies by Fernández-Tschieder, Binkley, and Bauerle [52] and Moreau et al. [53], which observed that trees in the upper canopy layers (dominant and codominant classes) generally exhibit faster growth due to better resource availability. In contrast, suppressed trees have slower growth rates as a result of reduced light and nutrient availability, which aligns with the principles of light competition and canopy stratification. In forest management, recognizing the differences in growth rates across crown classes is essential. Strategies such as selective thinning to remove suppressed and intermediate trees could help promote the growth of dominant and codominant trees, thereby improving overall forest productivity and maintaining a healthy forest structure [54, 55].

The diameter growth by quality class further highlights the importance of tree health and vigor in determining growth rates (Table 6 and Figure 5). High quality tree, characterized by optimal health, strong form, and minimal damage, showed the highest growth rates, with an average of 0.319 cm per year. These trees are likely to have fewer growth constraints, such as disease, pest infestation, or physical damage, allowing them to grow more rapidly than lower-quality trees. Trees under quality 2 had a slightly lower mean growth rate of 0.279 cm per year, likely due to moderate health issues or some competition, but still showing relatively strong growth compared with lower-quality trees. Crown quality 3 trees, with an average growth rate of 0.234 cm per year, exhibited the slowest growth (Table 6). These trees may be affected by poor health, structural damage, or unfavorable growing conditions, which restrict their ability to accumulate biomass and grow at a fast rate. These results are consistent with findings by Kröber et al. [56], Li et al. [57], and Madsen et al. [58] who supports the fact that high crown quality trees tend to grow faster due to fewer stress factors, while low-quality trees face growth limitations due to various biotic and abiotic factors. The relationship between tree crown quality and growth is critical for forest management strategies, as high-quality trees should be prioritized for conservation and future harvesting, while lower-quality trees may be targeted for thinning to improve the overall health and growth of the stand.

### 4.2. Diameter Growth Modeling of *T. alata*

The analysis of tree diameter growth using various models, including the generalized additive model, linear mixed model, and multiple linear model, provides insights into the factors influencing growth patterns (Tables 7, 8, 9, 10, 11, and 12). Both the linear mixed model and the multiple linear model showed that only DBH was a significant predictor, though these models had low explanatory power, with adjusted *R*^2^ values of 0.23 and 0.03, respectively. This limited accuracy suggests that tree diameter growth is influenced by more complex, nonlinear relationships that are not well captured by simple linear models [59]. This indicates that these models may be insufficient for datasets where variables interact in nonlinear ways, as is often the case in ecological studies [60]. In contrast, the generalized additive model, which achieved a higher adjusted *R*^2^ of 0.32, offers a more nuanced understanding by allowing for nonlinear effects, as evidenced by the significant smooth terms for multiple predictors (Table 11). In the generalized additive model, crown cover, height, DBH, and elevation were all significant predictors, revealing complex and variable-specific impacts on diameter growth. For example, the significance of crown cover as a smooth term suggests that its influence on growth is nonlinear, likely following an optimal range where neither too sparse nor too dense cover maximizes growth. This reflects real-world forest dynamics, where trees in intermediate canopy densities might experience favorable conditions, such as optimal light availability and nutrient competition, leading to increased growth rates [61]. Similarly, height and DBH show nonlinear relationships, implying that growth rates fluctuate at different stages of tree development, with younger, smaller trees often growing rapidly as they establish and mature, whereas older, larger trees grow more slowly due to aging and resource limitations [62]. These significant smooth terms suggest that interventions, such as thinning or managing crown cover, could be most effective if tailored to the nonlinear growth patterns of specific tree characteristics rather than using uniform strategies across the forest. Forest managers may achieve improved growth rates by adapting management practices to specific DBH classes, thus optimizing resources and improving forest productivity.

The low adjusted *R*^2^ values for the linear mixed model and multiple linear regression highlight the need for more sophisticated modeling techniques when dealing with complex ecological data. It is clear from this analysis that the generalized additive model offers superior model fit and a better understanding of the nonlinear relationships between the predictors and DBH growth. However, the overall accuracy of the models could still be improved. It is recommended that future studies consider incorporating additional variables that might explain more of the variation in tree growth, such as soil characteristics, moisture levels, or stand density [63–65]. Further refinement of model selection processes and the inclusion of interactions between predictors could also help enhance the accuracy of the predictions. In addition, larger and more diverse datasets could potentially strengthen the model's predictive power and allow for more generalizable conclusions. Growth models provide a reliable tool for evaluating silvicultural and harvesting choices, determining sustainable timber output, and analyzing the effects of forest management and harvesting on other forest values, given sufficient inventory and resource data [66]. Accurate predictions of current resource levels and expected resource changes resulting from specific management alternatives are necessary for developing tools that support informed decision-making in sustainable forestry [67].

## 5. Conclusions

This study underscores the importance of diameter, crown, and quality class distinctions in understanding growth dynamics of *T. alata*. The models used to predict diameter growth of *T. alata*, including the multiple linear model, linear mixed model, and generalized additive model, showed limited explanatory power but provided an initial framework for understanding the relationship between growth and available variables. This study indicated that some tree variables, such as crown cover, tree, height, and DBH, have complex, nonlinear effects on the diameter growth, and their relationships should be modeled flexibly. Further refinement of growth models using additional variables or data or even exploring new model alternative could improve the predictive accuracy.

## Figures and Tables

**Table 1:** Descriptive statistics of tree level and plot level attributes used in the study.

Attributes	Min	Mean	Max	St. deviation	Variance
DBH (cm)	5.1	38.8	119	23.9	571.1
Height (m)	1.4	19.3	43	9.1	82.6
DEM (m)	114	477.4	1453	301.5	90.69
Longitude (°)	80	81.9	85.1	1.4	1.9
Latitude (°)	27	28.3	29	0.5	0.3

**Table 2:** Number of sample trees falls within each quality class and crown class.

Crown class							
Quality class	1 (dominant)	2 (codominant)	3 (intermediate)	4 (suppressed)	5 (understory)	6 (broken)	Grand total
1	281	55	1		0	13	350
2	43	37	8	1	0	6	95
3	11	24	10	7	0	28	80
Grand total	335	116	19	8	0	47	525

**Table 3:** Summary of first and second period.

Annual DBH growth (cm·yr^−1^)	Min	1^st^ quartile	Median	Mean	3^rd^ quartile	Max
1^st^	−0.03	0.13	0.23	0.29	0.40	1.86
2^nd^	0.02	0.10	0.22	0.29	0.40	1.60

**Table 4:** Mean diameter growth rates and tree counts by DBH class (cm).

DBH class (cm)	Avg. diameter growth (cm·yr^−1^)	No. of trees
0–25	0.318	166
26–50	0.263	211
51–75	0.311	100
76–100	0.281	20
101–125	0.318	14

**Table 5:** Crown class wise description of DBH, height, and growth of trees.

Variables	Crown class	No. of trees	Min	Mean	Max	Std. dev	Var
DBH (cm)	1	335	5.3	47.37	114.8	20.98	440.09
	2	116	5.2	21.54	51.5	11.76	138.41
	3	19	5.1	13.16	34.5	7.15	51.14
	4	8	6.5	14.4	30.8	10.33	106.67
	6	47	5.2	34.27	118.6	35.29	1245.47
	Total	525	5.1	38.75	118.6	23.9	571.08

Height (m)	1	335	4.2	23.25	43	7.83	61.33
	2	116	2.7	14.39	28.7	5.41	29.32
	3	19	4.1	10.34	23	5.3	28.06
	4	8	4.9	10	18.6	5.6	31.36
	6	47	1.4	8.79	32.4	8.44	71.25
	Total	525	1.4	19.33	43	9.09	82.59

Growth (cm·yr^−1^)	1	335	0.017	0.303	1.867	0.271	0.074
	2	116	0.017	0.298	1.083	0.233	0.054
	3	19	0.017	0.181	0.4	0.124	0.015
	4	8	0.02	0.184	0.483	0.156	0.024
	6	47	0.017	0.263	1.067	0.25	0.063
	Total	525	0.017	0.292	1.867	0.257	0.066

**Table 6:** Quality class-wise description of DBH, height, and growth of trees.

Variables	Quality class	No. of trees	Min	Mean	Max	Std. dev	Var
DBH (cm)	1	350	5.2	46.32	118.6	23.08	532.79
	2	95	6.5	28.75	82.2	15.58	242.61
	3	80	5.1	17.5	113.4	17.72	313.92
	Total	525	5.1	38.75	118.6	23.9	571.08

Height (m)	1	350	1.7	23.03	43	7.81	60.93
	2	95	2.1	15.16	33.7	5.73	32.81
	3	80	1.4	8.09	20.7	5.49	30.16
	Total	525	1.4	19.33	43	9.09	82.59

Growth (cm·yr^−1^)	1	350	0.02	0.31	1.87	0.27	0.07
	2	95	0.02	0.26	1.42	0.23	0.05
	3	80	0.02	0.26	1.07	0.21	0.04
	Total	525	0.02	0.29	1.87	0.26	0.07

**Table 7:** DBH growth rate in multiple linear model.

Min	1^st^ quartile	Median	3^rd^ quartile	Max
−0.31775	−0.16178	−0.04858	0.09634	1.50261

**Table 8:** Summary table of multiple linear model.

Parameter	Coefficient	Std. error	*t*-value	*p* value
Intercepts	2.156e + 00	4.892e + 00	0.411	0.6597
DBH (cm)	−2.046e − 03	9.114e − 04	−2.245	0.0254
Height (m)	4.11e − 03	3.029e − 03	1.457	0.1461
Crown height	2.742e − 03	4.519e − 03	0.607	0.5444
Crown cover (%)	−2.491e − 04	8.804e − 04	−0.283	0.7774
DEM (m)	−8.310e − 05	4.831e − 05	−1.720	0.0862
Longitude (°)	−2.136e − 02	4.138e − 02	−0.681	0.4966
Latitude (°)	−3.214e − 03	8.445e − 02	−0.038	0.9697

*Note:* Residual standard error: 0.2661 on 896 degree of freedom. The equation obtained from this model can be expressed as follows: growth = *a* + *b* ∗ DBH + *c* ∗ height + *d* ∗ crown height + *e* ∗ crown cover + *f* ∗ dem + *g* ∗ longitude + *h* ∗ latitude. In this equation, the coefficients *a*, *b*, *c*, *d*, *e*, *f*, *g*, and *h* represent the respective parameters as shown in the previous Table 11.

**Table 9:** Summary table for fixed effects of the linear mixed model.

Parameter	Coefficient	S.E.	95% CI	*t* (370)	*p* value
Intercepts	0.65	5.47	[−10.09, 11.40]	0.12	0.905
DBH (cm)	−2.34e − 03	9.67e − 04	[−0.00, 0.00]	−2.42	0.016
Height (m)	4.93e − 03	3.16e − 03	[−0.00, 0.01]	1.56	0.120
Crown height (m)	1.76e − 03	4.67e − 03	[−0.01, 0.01]	0.38	0.707
Crown cover (%)	1.46e − 03	9.11e − 04	[−0.00, 0.00]	0.16	0.873
DEM (m)	−7.85e − 05	5.60e − 05	[−0.00, 0.00]	−1.40	0.162
Longitude (°)	−0.01	0.03	[−0.08, 0.06]	−0.34	0.737
Latitude (°)	0.02	0.09	[−0.16, 0.21]	0.23	0.821

*Note:* 95% confidence intervals (CIs) and *p* values were computed using a Wald *t*-distribution approximation. The resulting equation from this model is growth = *a* + *b* ∗ DBH + *c* ∗ height + *d* ∗ crown ht + *e* ∗ crown cover + *f* ∗ dem + *g* ∗ longitude + *h* ∗ latitude + *i* ∗ plot no + *j* ∗ residual. In this equation, the coefficients *a*, *b*, *c*, *d*, *e*, *f*, *g*, and *h* represent the respective parameters.

**Table 10:** Summary table for random effect of linear mixed model.

Parameters	Coefficients	S.E	95% CI
Intercept: plot no	0.11	0.02	[0.08, 0.16]
Residual	0.22	0.01	[0.20, 0.24]

**Table 11:** Generalized additive model result for diameter growth.

Parametric coefficients:					
Variables	Estimate	Std. error	*t*-value	Pr (> |*t*|)	Sig
(Intercept)	0.29554	0.04284	6.899	1.57E − 11	⁣^∗∗∗^
Factor (time) 2022	0.08122	0.05557	1.461	0.145	

**Approximate significance of smooth terms**					
**Smooth terms**	**edf**	**Ref. df**	***F* value**	**p** **value**	**Sig**

s (crown_height)	1	1	1.829	0.17682	
s (crown_cover)	7.969	8.684	4.5	1.23E − 05	⁣^∗∗∗^
s (height)	2.502	3.208	3.942	0.00743	⁣^∗∗^
s (DBH)	8.951	8.999	21.057	< 2e − 16	⁣^∗∗∗^
s (dem)	8.322	8.865	2.971	0.0013	⁣^∗∗^
s (plot_Id)	1.618	5	0.484	0.1872	

*Note:* Parametric coefficients include estimates, standard errors (std. error), *t*-values (*t* value), and corresponding *p* values indicating the statistical significance of each predictor variable. Smooth terms are presented with effective degrees of freedom (edf), reference degrees of freedom (Ref. df), *F*-statistics (F), and associated *p* values. Significance codes in the table are interpreted as follows: *p* < 0.001 is denoted by “⁣^∗∗∗^”, *p* < 0.01 by “⁣^∗∗^”, and *p* < 0.05 by “⁣^∗^”.

**Table 12:** Summary of generalized additive models (GCV means generalized cross validation).

Model	*R*-squared (adj)	Deviance explained (%)	GCV	Scale estimation
GAM	0.326	36.60	0.38961	0.36617

## Data Availability

The data used to support the findings of this study are available on request from the corresponding author.
